# A241 CORRELATION BETWEEN PLASMA IGA LEVEL AND BIOLOGICAL THERAPY IN PATIENTS WITH INFLAMMATORY BOWEL DISEASE

**DOI:** 10.1093/jcag/gwad061.241

**Published:** 2024-02-14

**Authors:** B Lee, J Hsu, T Tsai

**Affiliations:** School of Medicine, China Medical University, Taichung, Taiwan, Taichung, Taiwan; Center for Translational Genomics & Regenerative Medicine Research, China Medical University Hospital, Taichung, Taiwan., Taichung, Taiwan; Center for Digestive Medicine, Department of Internal Medicine, China Medical University Hospital, Taichung, Taiwan, Taichung, Taiwan

## Abstract

**Background:**

IgA is the predominant antibody class secreted by intestinal immune cells. However, the level of plasma IgA in Inflammatory Bowel Disease (IBD) patients under medical treatment remains unknown. Therefore, our study aimed to investigate the relationship between plasma IgA concentration in IBD patients under biological therapy.

**Aims:**

To assess the correlation between plasma IgA level in IBD patients under biological therapy.

**Methods:**

We collected data from outpatient clinics at our hospital, including 12 IBD patients, comprising 4 patients with Crohn's disease (CD) and 8 patients with Ulcerative Colitis (UC). Data was collected between 2020 and 2023. We measured plasma IgA concentrations both before initiating biologic therapy and at the 14th week of biological treatment (4 under Adalimumab and 8 under Vedolizumab). Additionally, we compared baseline plasma IgA levels between 24 IBD patients (7 CD and 17 UC) and 24 healthy individuals. Student T test was used for statistical analyze.

**Results:**

The average baseline plasma IgA level was significantly higher in IBD patients (2134±187.2 ug/ml, mean ± standard error) when compared to healthy individuals (1346± 114.8 ug/ml, p=0.0008). In the view of CD and UC, the baseline plasma IgA level was 2179±439.4 ug/ml and 2116±203.1 ug/ml, respectively. Baseline plasma IgA level also showed statistical significantly higher in CD and UC compared to healthy individuals (p=0.0063, p=0.0005, respectively), and without difference between UC and CD patients (p=0.8824) (Figure 1A).

Furthermore, we investigated the impact of biological therapy on plasma IgA levels in IBD patients, finding a significant decrease in plasma IgA levels after biological therapy (2182±254.7 ug/ml to 1824±135.5 ug/ml, p=0.0181). Similar trend was noted in UC patients under biological therapy with borderline statistical significant (2141± 307.7ug/ml to1809±173.3ug/ml, p=0.0561), but not found in CD patients (2265±518.3 ug/ml to 1855±247.5 ug/ml, p= 0.1333)(Figure 1B).

**Conclusions:**

We found that baseline plasma IgA level was higher in IBD patients compared to healthy individuals. Besides, plasma IgA level decreased after biological therapy in IBD patients, especially in UC patients. These findings suggest that plasma IgA level may be a potential biomarker for IBD patients.

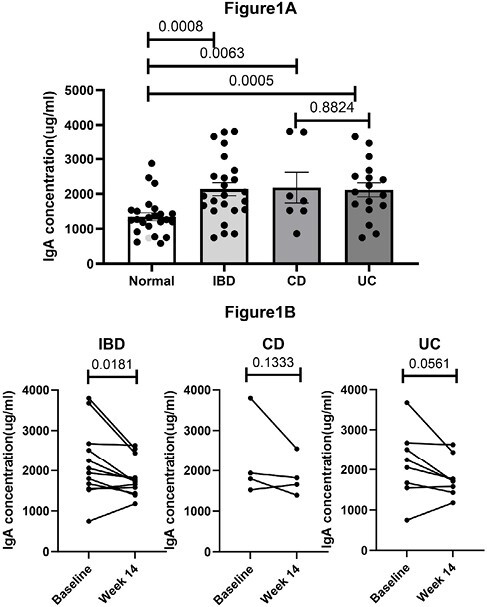

**Funding Agencies:**

None

